# Myocardial Infarction Presenting as Ear Fullness and Pain

**DOI:** 10.1177/2324709618761753

**Published:** 2018-03-09

**Authors:** Israel Ugalde, Ibrar Anjum, Saberio Lo Presti, Alfonso Tolentino

**Affiliations:** 1 Mount Sinai Medical Center, Miami Beach, FL, USA; 2 Akhtar Saeed Medical and Dental College, Lahore, Pakistan; 3 Columbia University, Mount Sinai Medical Center, Miami Beach, FL, USA

**Keywords:** myocardial infarction, ear pain, otalgia, ear fullness, NSTEMI

## Abstract

Acute coronary syndrome usually presents with retrosternal chest pain, nausea, vomiting, sweating, and jaw and arm pain. Some patients only present with neck, epigastric, or ear discomfort. A 47-year-old male with a history of hypertension and coronary artery disease presented to the emergency department complaining of bilateral otalgia. He never felt chest pain, jaw pain, nausea, diaphoresis, or shortness of breath. He had a history of 2 acute coronary events and had a stress test 2 months prior to admission, which was unremarkable. The initial electrocardiography was sinus rhythm with Q-waves in the inferior leads and nonspecific ST changes in the lateral leads. His troponin on admission was normal but subsequently elevated to 20.00 mg/mL after 24 hours. He underwent left heart catheterization, which found significant occlusive disease of the second and fourth obtuse marginal branches and 2 drug-eluting stents were placed. His ear pain resolved soon after cardiac catheterization. The pathophysiology of this referred pain is thought to be related to the neuroanatomy of the nerves innervating the heart and ear. The auricular nerve branch of the vagus nerve supplies the inner portion of the external ear. Only a few cases with the complaint of otalgia have been reported. Patients were older, more frequently women, and with diabetes or heart failure. Clinicians should be aware of the atypical presentation of angina that may be life-threatening cardiac ischemia. Ear pain and fullness could be the sole presenting symptom in a patient with acute coronary syndrome.

## Introduction

Heart disease is the leading cause of death in the United States and around the world.^1^ In the realm of heart disease, mortality includes acute coronary syndrome (ACS). ACS is a spectrum of heart diseases that include ST-segment elevation myocardial infarction, non-ST-segment elevation myocardial infarction, and unstable angina.^2^ ACS usually presents with pressure-like retrosternal chest pain, nausea, vomiting, sweating, and jaw and arm pain. Some patients only present with neck, epigastric, or ear discomfort.^2^ Craniofacial pain can present as the sole symptom in up to 6% of patients with myocardial infarction.^3^ Described here is a case of ACS presenting with atypical symptoms solely as bilateral ear fullness and ear pain.

## Case Presentation

**Figure 1.**
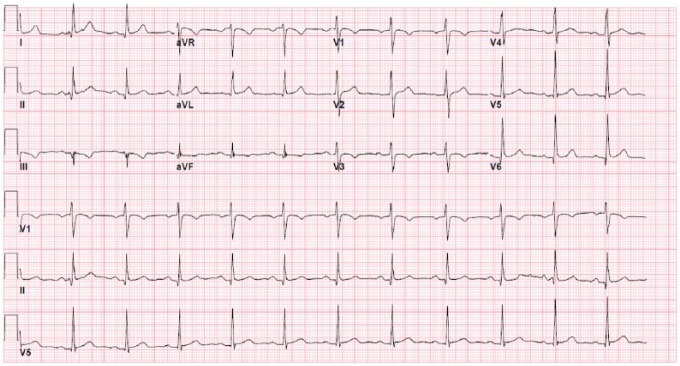
Admission EKG with Q-waves in the inferior leads and nonspecific ST changes in the lateral leads.

A 47-year-old male with a past medical history of hypertension and coronary artery disease presented to the emergency department complaining of bilateral otalgia. He woke up in the morning and was about to have breakfast when his symptoms suddenly started. The bilateral ear pain lasted approximately 30 to 45 minutes, and the pain did not radiate and was only mildly improved after he took his home medications, which included aspirin and a β-blocker. The persistence of his symptoms prompted his visit to the emergency department. Pertinent negatives include no chest pain, shortness of breath, nausea, vomiting, diaphoresis, dizziness, tinnitus, hearing loss, fevers, or chills. His past medical history was significant for 2 non-ST-elevation myocardial infarctions 6 and 10 years prior to admission that required 3 coronary stents, which he did not know any additional information about. He was compliant with his medications including bisoprolol, atorvastatin, and aspirin therapy daily. He denied any alcohol or tobacco use. His father had a history of coronary artery disease in his 40s. He had a stress test 2 months prior to admission that was unremarkable for any ischemic electrocardiography (EKG) changes. On arrival to the emergency department, his physical examination revealed that he was afebrile and hemodynamically stable. He appeared comfortable in no acute distress. His cardiovascular examination exhibited a normal rate and regular rhythm with no murmur or friction rub. Otoscopic examination was unremarkable with no discharge, tympanic swelling, or loss of light reflex. The initial EKG showed sinus rhythm with Q-waves in the inferior leads and nonspecific ST changes in the lateral leads (Figure 1).

**Figure 2.**
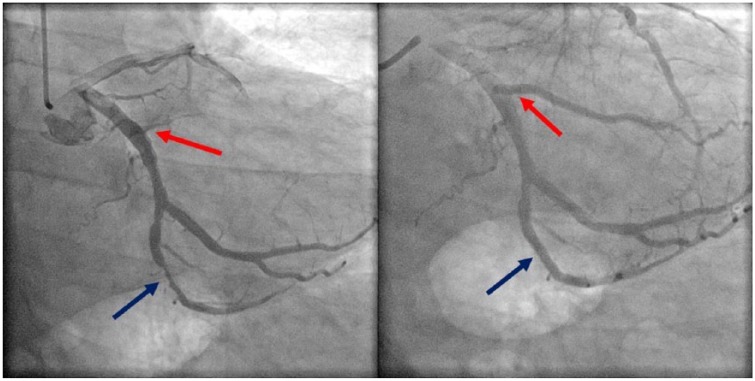
Second obtuse marginal (red arrow) and fourth obtuse marginal (blue arrow) arteries before (left) and after (right) DES.

He was admitted to the hospital for observation given his significant history of coronary artery disease. His initial troponin was normal but subsequently rose to a peak of 20.00 mg/mL after 24 hours. A transthoracic echocardiogram was interpreted as having an ejection fraction between 60% and 65% with no obvious valvulopathy or wall motion abnormalities. While his troponins were increasing, repeat EKGs showed development of sinus bradycardia at a rate of 48 beats per minute with no significant ST-segment changes. Because of the significant elevation in cardiac biomarkers, he underwent left heart catheterization, which revealed significant occlusive disease of the second and fourth obtuse marginal branches (Figure 2). Two drug-eluting stents (Boston Scientific SYNERGY 2.75 × 11 mm and 3.0 × 24 mm) were placed in the second and fourth obtuse marginal arteries, respectively (Figure 2). Luminal irregularities were also noted in the left anterior descending artery, diagonal branches, left circumflex artery, and dominant right coronary artery. His ear fullness and otalgia improved immediately after the angioplasty and completely resolved the following day.

## Discussion

Otalgia is reported as a possible presentation of ACS in the 2014 American Heart Association guidelines for management of non-ST-elevation myocardial infarction.^2^ Somatic referred pain is due to a nerve stimulus at a site other than the noxious stimulus. The pain is commonly described as dull, aching, and gnawing. There is no weakness or neurological symptoms because there is no compression of the spinal nerves.^4^ The pathophysiology is thought to be related to the neuroanatomy of the nerves innervating the heart and ear. The sensory innervation of the ear is a combination of cervical and cranial nerves.^5^ All of these nerves enter the spinal tract nucleus near the medulla. The stimulation of any of these nerves can cause referred ear pain based on the convergence-projection theory.^5^ This theory suggests that the central nervous system cannot differentiate between stimuli that converge on common sensory pathways.

The vagus nerve innervates the sinoatrial (SA) node and atrioventricular (AV) node in the heart. The auricular nerve branch of the vagus nerve, also known as Arnold’s or Alderman’s nerve, supplies the inner portion of the external ear. Stimulation of the auricular branch of the vagus nerve can lead to referred otalgia as described in the case report. The autonomic input of the heart has been previously described with a certain degree of laterality. While the right SA node receives innervation from the right vagus nerve, the AV node is innervated mainly by the left vagus and sympathetic nerves. The interaction is complex and significant overlap can occur.^6^

Any occlusion of right coronary artery can result in decreased blood supply and damage to the vagus nerve. This can lead to autonomic dysfunction of the auricular branch of the vagus nerve producing ear fullness and referred pain in the ears.^7,8^ However, the arterial supply to the SA node comes from the right coronary in 55% to 60%, or the left circumflex artery in 40% to 45% of patients. The blood supply can approach the SA node in a clockwise or counterclockwise direction around the superior vena cava into the right atrial junction.^6^ There are rare cases that describe a dual blood supply to the SA node that could account for our patient’s unique presentation of bilateral ear pain.^9^ This is further supported by the observation that in the hours prior to cardiac catheterization, he developed sinus bradycardia. This could have been because of compromised collateral blood supply to the SA node arising from obstructive disease of the left circumflex artery. ST-segment changes were not noted in subsequent EKGs but posterior leads V7 to V9 were not obtained.^10^

Only a few cases with the complaint of otalgia but not with ear fullness have been reported.^7,11-15^ A review of literature found the age of presentation was above 50 years with delayed diagnosis as other conditions were higher on the differential. Patients commonly had comorbidities such as heart failure or diabetes. Non-ST-segment elevation myocardial infarction and unstable angina were the most common final diagnosis. Only one reported case of ST-segment elevation myocardial infarction presenting as otalgia was reported.^12^ This is consistent with reports of myocardial infarction presenting with atypical chest pain symptoms in patients who are older, more frequently women and with diabetes or heart failure.^16^

## Conclusion

This case highlights an important diagnostic challenge for the general practitioners and emergency room physicians. Delay in the diagnosis of ACS has been associated with poor clinical outcomes and increased mortality. Clinicians should be aware of the atypical presentation of angina that may be life-threatening cardiac ischemia. Ear pain and fullness could be the sole presenting symptoms in certain patients with ACS. These atypical symptoms should be included in the evaluation of patients suspected of having acute cardiac ischemia.
